# Supplementary material for: Are carbon emissions trading and green financial instruments synergistic? -Comprehensive quantitative research based on content analysis
Source: PLoS One. 2024 Mar 7;19(3):e0298601. doi: 10.1371/journal.pone.0298601 (PMC10919601; doi:10.1371/journal.pone.0298601)
Supplement: S1 Data — (ZIP) [file pone.0298601.s001.zip › Data/地级市Z_F工作报告.zip!/╔╜╢1⁄2/╠⌐░▓╩╨/└┤╫╘╣1⁄2╓┌║┼í╛┬φ┐╦╩2╛▌═°í┐_2012.docx]

各位代表：

　 现在，我代表泰安市人民政府向大会报告工作，请予审议，并请各位政协委员和其他列席会议的同志提出意见。

　　一、攻坚克难，全市经济与社会保持平稳较快发展

　　2008年以来，是泰安发展历程中极不平凡的时期。面对国内外复杂形势和一系列重大风险挑战，在中共泰安市委的领导下，在历届班子打下的良好基础上，全市上下认真贯彻党的十七大和十七届历次全会精神，坚持以科学发展观统揽全局，解放思想，科学务实，积极作为，攻坚克难，圆满完成了“十一五”规划，实现了“十二五”规划良好开局，全市改革开放和现代化建设取得显著成绩。

　　(一)经济综合实力明显增强。2011年全市实现生产总值2304.3亿元，年均增长13.1%。其中一二三产业增加值年均分别增长3.8%、12.4%和16.6%；完成地方财政收入138.1亿元，年均增长21.1%；实现社会消费品零售额806.7亿元，年均增长19.6%；金融机构各项存、贷款余额达到1563.6亿元、1043.2亿元，年均增长17.8%和17.9%。

　　(二)转方式调结构加快推进。三次产业比例由2007年的10.8：56.6：32.6调整为9.3：52.2：38.5。现代农业加快发展，农业综合生产能力和产业化水平明显提高。去年粮食总产达到317.1万吨，实现连续九年增产，其他农产品产量和质量都有较大提高，东平县获“全国粮食生产先进单位”称号；有机蔬菜、奶牛养殖、苗木花卉、桑蚕生产等走在全省乃至全国前列；规模以上农业龙头企业达到428家，农民专业合作社发展到2769家。认真落实各项强农惠农政策，累计发放粮食直补、良种补贴、农机补贴等资金17亿元。工业结构不断优化，主导产业、骨干企业、知名品牌培植取得明显成效，八大主导产业调整振兴、战略性新兴产业发展势头良好。全市规模以上工业企业达到1560家，比2007年增加555家；主营业务收入、利税、利润分别达到4550.2亿元、579.5亿元、361.3亿元，年均增长24.8%、27.5%和29.8%；其中主营业务收入过10亿元、实缴税金过亿元的企业达到66家和12家，增加40家和6家。中国名牌产品、中国驰名商标达到6个和20件。以旅游文化产业为龙头的服务业快速发展。全市服务业增加值886.5亿元，年均增长16.6%；去年泰山游客达到459.3万人，年均增长16.1%；旅游总收入达到318.7亿元，年均增长30.6%；文化产业增加值占生产总值的比重预计达到4.2%，提高1.3个百分点。创新型泰安建设加快推进，高新技术产业产值占规模以上工业总产值的比重达到21.3%，提高1.2个百分点，国家高新技术企业发展到59家，获得省级以上科学技术奖励231项，泰安跻身“中国城市综合创新力50强”，荣获“全国科技进步先进市”称号。节能减排取得明显成效，万元GDP能耗、规模以上工业万元增加值能耗、二氧化硫和COD排放量持续下降，全面完成省下达的节能减排任务。土地节约集约利用水平明显提升，耕地得到有效保护。创建国家环保模范城取得积极进展，顺利通过省级预评估。生态环境明显改善，全市森林覆盖率达到37.8%。

　　(三)项目建设成效显著。累计完成固定资产投资4632.7亿元，年均增长23.8%；开工建设亿元以上的重点项目687个，投资总规模3443亿元；争取中央扩大内需项目226个、4.1亿元，省调控资金项目53个、11亿元。新开工总投资3000万元以上的国内招商引资项目2031个，到位资金1064亿元。投资结构逐步优化，其中高新技术产业和第三产业投资年均增长30.4%和33.3%。青年汽车、山东煤机工业园、特变电工华东产业园、塔高矿山机械、泰山方特欢乐世界、泰山封禅大典大型实景演出、水浒影视基地等一大批投资过亿元的项目竣工投入运营；航天特车工业园、20万吨石膏板护面纸、中青旅泰山龙曦国际旅游度假地等一批投资过亿元的项目正在加紧建设。

　　(四)改革开放迈出新步伐。国有资产监管和组织管理体系进一步完善，企业改革改制不断深化，国有资产实现保值增值。境内外上市企业达到7家。农村综合配套改革继续深化，土地流转制度改革稳步推进，集体林权制度主体改革圆满完成。新一轮政府机构改革顺利完成。文艺院团、出版发行等文化领域体制改革进展顺利，荣获“全国文化体制改革工作先进市”称号。财税体制、事业单位、医药卫生体制等改革取得新进展。累计新批利用外资项目572个，实际利用外商直接投资4.65亿美元，完成进出口贸易总值59.9亿美元，其中出口37.4亿美元。培育省级出口名牌4个、科技兴贸出口创新基地3家；对外交流合作进一步扩大，与14个国家的18个城市建立了友好合作关系。

　　(五)城乡面貌显著变化。《泰安市城市总体规划(2011—2020年)》已经国务院批复，先后展开了“两轴”(历史文化轴、时代发展轴)、“两带”(环山带、泮河带)、“五大片区”等九大城市组团开发建设，基础设施和生态环境明显改善。京沪高铁泰安站及广场、泰山桃花峪和天外村游人服务中心、长城路北段综合开发、泰山火车站广场综合改造、旅游开发区道路等一批基础设施相继建成投入使用，泰山报业文化中心、泰山会展中心等项目建设进展顺利。泰山环山路荣获建筑业界最高奖项“鲁班奖”。泰城新增城区面积940万平方米、道路247万平方米、公园绿地267万平方米，污水集中处理率提高到91%，用水普及率和城市垃圾无害化处理率均达到100%，全市城镇化率达到51.4%，提高4.8个百分点。城市环境综合整治成效显著，居住环境和居民生活质量明显提升。各县市区驻地和小城镇建设也都实施了一批重点工程项目，城镇综合服务功能不断增强。农村社区建设和危房改造全面展开，“路、水、电、医、学”建设进一步加强。泰城、新泰、肥城先后被命名为“国家园林城市”，宁阳、东平已通过验收；泰安获得“国家节水型城市”称号，通过国家卫生城市复审。

　　(六)社会事业繁荣发展。城乡免费义务教育全面实现，高中阶段教育基本普及，职业教育、成人教育、高等教育持续发展。学前三年教育入园率达到91.6%，高中阶段入学率达到96.5%，驻泰高校在校生达到9.8万人，在省教育督导中连续名列前茅。“人才强市”战略顺利实施，专业技术、高技能人才引进培养取得明显成效。公共文化服务体系进一步完善，87个乡镇(街道)全部建成了综合文化站，国家级重点文物保护单位9处、非物质文化遗产名录9项。城乡医疗卫生条件不断改善，新建改建城市社区卫生服务机构181处、乡镇中心卫生院74个、村卫生室2692个。奥运金牌实现零突破，奥运火炬传递、全运圣火采集和赛事承办圆满成功，公共体育设施建设得到改善，群众体育广泛深入开展。人口继续保持低生育水平，年均人口自然增长率为4.06‰，先后荣获“全国婚育新风进万家活动示范市”、“全国人口和计划生育综合改革示范市”称号。第六次人口普查圆满完成。群众性精神文明创建活动深入开展，民主法制建设得到加强。深化“平安泰安”建设，严密防范、严厉打击违法犯罪活动，重视加强信访和社会矛盾化解工作，强化安全生产和食品药品安全整治，扎实开展社会管理创新全国综合试点工作，社会保持和谐稳定。圆满完成四川地震灾区援建任务，对口支援新疆岳普湖县、扶贫协作重庆巫溪县取得积极成果。国防动员、人防建设、民兵预备役、双拥共建等取得新成绩。妇女儿童和残疾人保障、老龄工作及服务有新的提高。外事侨务、民族宗教、爱国卫生、气象、防震、史志、档案等社会事业全面进步。先后荣获“全国双拥模范城”、“全国民族团结进步模范集体”等称号。

　　(七)民生得到有效保障。城镇居民人均可支配收入、农民人均纯收入分别达到22687元和8974元，年均增长13.2%和14%。全市用于教育、社会保障和就业、医疗卫生、农林水事务、住房保障等民生事业财政支出累计323.8亿元，占财政总支出的比重达到51.4%。累计新增城镇就业再就业28.5万人，农村劳动力转移就业32.7万人，城镇登记失业率控制在3.5%以内。覆盖城乡的社会保障体系基本建立，社会保障水平不断提高，各项社会保险收支总规模达到391.6亿元。企业退休人员月人均基本养老金增加566元。城镇居民医保制度全面建立，城镇职工、居民医保和新农合报销比例提高到75.2%、70.1%和70%以上，城乡居民社会养老保险提前一年实现全覆盖。加快机构养老福利服务设施建设，实施社会散居孤儿、残疾儿童福利制度，新建和改扩建敬老院83所，集中供养率达到76.6%，基本实现应保尽保。连续4次提高城乡最低生活保障标准，低保人数由5.7万人扩大到18.7万人。累计归集住房公积金67.8亿元，发放贷款45.7亿元。加大保障性住房建设力度，累计建设廉租住房3876套、经济适用住房8669套和公共租赁住房4574套。495个村启动了整村迁建，建设农房18万户，完成危房改造4.5万户。实施公交优先战略，泰城更新环保型公交车102台，公交网络覆盖面积增加10.8%。村村通柏油路、通客车率达到99.9%和99.5%。全面完成12座大中型、426座小型病险水库除险加固任务；累计投资2.93亿元，解决了58.6万农村人口吃水困难，农村自来水普及率达到98%。完成农村无害化生态卫生厕所1.5万座，新建户用沼气池4.9万个。

　　(八)政府施政水平明显提升。大力推进政府职能转变，进一步完善行政审批、政府采购、应急管理等政务服务功能和运行机制，全面推行行政服务标准化，政府运行更加规范高效。全面清理行政许可和非行政许可事项，减少市级行政审批311项，集中清理规范性、政策性文件2154件，废止722件、修改123件。积极推行并联审批和标准化服务，审批服务质量和效率进一步提高。便民服务电话、民生直通车网站建设与服务水平进一步提升，电子政务、政务公开、政风行风评议深入开展。认真落实《公务员法》，大力加强公务员队伍建设，干部队伍素质和施政能力有了新的提高，发展环境进一步优化。

　　各位代表，上述成绩的取得，来之不易。这是在市委坚强领导下，全市人民团结奋斗，开拓进取，顽强拼搏，真抓实干的结果；也是驻泰各单位和各界人士全力支持、自觉融入泰安经济与社会发展的结果。在此，我代表泰安市人民政府，向全市广大干部群众、各民主党派、工商联、无党派爱国人士，向人民解放军和武警驻泰部队及驻泰单位，向关心支持泰安发展的港澳台同胞、海外侨胞及海内外朋友，表示崇高的敬意和衷心的感谢！

　　在充分肯定成绩的同时，我们也清醒地看到发展中还存在不少困难和问题，政府工作也有许多不足。一是经济综合实力还不强，产业结构还不合理，工业主导产业优势不明显，现代服务业发展不快，骨干企业较少、核心竞争力不强。二是经济发展的质量和效益有待提升，企业自主创新能力还不强，高新技术产业比较薄弱，节能降耗、环境保护压力较大，转变发展方式的任务繁重。三是改革开放的力度需进一步加大，体制性、机制性障碍依然存在，小微企业生产经营困难，外向型经济发展明显滞后。四是城市功能还需要进一步完善，城乡统筹任务艰巨。五是稳定物价和扩大消费需求压力较大，保障改善民生还有大量的工作要做，社会管理创新有待深入探索。六是政府工作人员的思想观念还需进一步解放和更新，行政效能和服务质量需要进一步提高，发展环境需要进一步优化等等。我们必须强化危机意识和忧患意识，把困难估计得更充分一些，把措施考虑得更周全一些，把工作做得更扎实一些，顽强拼搏，奋发进取，不断夺取推进富民强市、建设幸福泰安的新胜利。

　　二、科学谋划，促进泰安经济与社会事业科学发展、跨越发展、和谐发展

　　今后五年，是全面建设小康社会并向基本实现现代化迈进的关键时期，也是深化改革开放、推动转型升级的攻坚时期。政府工作的总体要求是：高举中国特色社会主义伟大旗帜，以邓小平理论和“三个代表”重要思想为指导，深入贯彻落实科学发展观，按照市第十次党代会要求，始终坚持以经济建设为中心，牢牢把握科学发展主题和加快转变经济发展方式主线，紧紧围绕推进富民强市、建设幸福泰安这个目标，以改革创新为动力，以项目建设为抓手，着力转型升级，着力优化环境，着力改善民生，全面加强经济建设、文化建设、社会建设以及生态文明建设，为加快实现全面小康社会而努力奋斗。政府工作总的预期目标是：

　　——综合实力显著提升。经济保持平稳较快发展，主要经济指标增速高于全省平均水平，人均主要指标力争达到或超过全省平均水平；地方财政收入平稳较快增长，保障能力明显增强，税收占财政收入的比重、主体税收占税收的比重明显提高。

　　——转方式调结构迈出大的步伐。经济结构进一步优化，形成具有泰安特色的现代产业体系。现代农业快速发展，产业化水平进一步提高；现代工业体系逐步形成，战略性新兴产业逐步崛起；现代服务业繁荣发展，旅游文化支柱产业地位巩固提升，服务业占经济总量的比重有较大提高；自主创新能力明显增强，高新技术产业产值占规模以上工业总产值的比重逐年提升。

　 ——城乡区域协调发展。城乡一体化进程明显加快，城镇化水平有较大提高，新农村建设取得新成效；泰城中心城市辐射带动能力明显增强；构建起经济支撑有力、基础设施完善、服务功能健全、人居环境优美、大中小城市和小城镇布局合理的现代化城镇体系。

　　——社会事业繁荣发展。加强基本公共服务体系建设，教育水平进一步提高，公共文化服务体系基本完善，城乡医疗卫生服务体系健全；切实稳定人口低生育水平；社会管理制度不断完善，社会更加和谐稳定。

　　——人民生活更加幸福。实现居民收入增长与经济发展同步，劳动报酬增长与劳动生产率提高同步；加大民生保障投入，就业再就业水平进一步提高，社会保障体系更加完善，人民生活更加美好幸福。

　　——生态环境优美宜居。单位GDP能耗、水耗、二氧化碳排放强度及化学需氧量、二氧化硫、氮氧化物和氨氮排放量等资源环境约束性指标达到或好于全省考核标准，森林覆盖率明显提高，生态环境质量不断改善。

　　实现上述预期目标，我们虽然面临一些困难，但更面临着机遇。我国目前仍处在可以大有作为的重要战略机遇期，经济增速在适度回落中逐步趋稳，为深化改革、转方式调结构提供了有利条件。近些年来，全市上下坚定不移地落实科学发展观，坚定不移地贯彻执行国家一系列宏观调控政策，坚定不移地抓项目建设和招商引资，特别是在应对国际金融危机挑战的实践中，积累了宝贵的经验，驾驭能力和领导水平有了新的提高，广大干部群众加快发展的信心、欲望更加坚定和高涨。只要我们审时度势，抢抓机遇，奋力拼搏，全市经济与社会就一定能够在现有基础上实现又好又快发展。在工作指导和把握上，要做到“五个坚持”：必须始终坚持科学发展。紧紧扭住发展第一要务不放松，加快推进转方式调结构，强化自主创新和节能减排，努力提高发展的质量、效益和可持续性；必须始终坚持改革创新。着力解决影响科学发展的体制性、结构性矛盾，加快推进重点领域和关键环节改革，推进体制机制创新和工作创新，使经济和各项事业发展始终充满生机和活力；必须始终坚持市委确定的战略目标。以推进富民强市、建设幸福泰安为目标，以项目建设和招商引资为抓手，丰富内涵，把握规律，提升标准，强力推进，创造发展的新优势；必须始终坚持统筹兼顾、重点突破。紧紧抓住牵动全局的重点工作、涉及群众利益的突出问题、影响发展的关键环节、事关全局的重点项目，强化措施，加大力度，力求实现大的突破，进而促进整体工作上水平；必须始终坚持以人为本。更加重视保障和改善民生，认真解决好人民群众最关心最直接最现实的利益问题，让人民群众共享改革发展成果，切实维护社会和谐稳定，充分调动广大人民群众的积极性、创造性，形成推进科学发展、跨越发展、和谐发展的强大合力。

　　三、励精图治，集中精力做好2012年的政府工作

　　2012年，是新一届政府的开局之年，做好今年的各项工作，意义深远，责任重大。按照市委的总体部署，与“十二五”规划相衔接，全市经济社会发展的主要预期目标是：生产总值增长11%；地方财政收入增长15%；规模以上固定资产投资增长18%左右；社会消费品零售总额增长16%；进出口总值增长12%左右；城镇居民人均可支配收入增长11%，农民人均纯收入增长12%；城镇新增就业再就业5万人，城镇登记失业率控制在3.5%以内；人口自然增长率控制在6.4‰以内；全面完成省政府下达的万元GDP能耗、二氧化碳排放强度及化学需氧量、二氧化硫、氮氧化物和氨氮排放量等节能减排任务。

　　(一)强力推进项目建设，增强经济社会发展的后劲与实力。牢固树立“没有项目就没有发展、没有大项目就没有跨越发展、没有好项目就没有科学发展”的理念，紧紧围绕增加财政收入、增加居民收入，大力实施“项目带动战略”，围绕三次产业协调发展和基础设施建设，引进、新上一批战略支撑项目、产业集群项目，为经济社会又好又快发展提供强力支撑。一是如期有效建项目。完善创新项目建设推进机制，协调解决项目建设中的困难和问题，实行跟踪问效，全力加快在建项目进度，确保如期达产达效，防止出现“半拉子工程”。对闲置和停建项目，实行分类指导，帮助企业走出困境、产生效益，避免资产闲置和资源浪费。年内，集中精力抓好中材玻纤新材料工业园、康平纳高速高效特种数控机床等100个重点项目建设。配合有关方面，加快山西中南部铁路通道、青兰高速公路、东平湖至济宁段调水与航运工程等国家、省重大基础设施项目建设。抓好国电泰安热电联产、中石油范镇天然气调峰等项目前期工作，力争早日批复开工。二是精心策划选项目。坚持高精尖标准，立足我市资源禀赋、产业基础和比较优势，认真研究国家宏观政策、产业导向和投资需求，围绕优势传统产业改造提升、战略性新兴产业培育、生产性服务业发展、旅游文化产业开发和国内外产业转移承接，精心策划包装一批环境污染少、经济效益好、带动能力强的重大战略性项目，特别注重从产业链的角度精选项目。全年策划总投资过亿元的项目80个以上。推进泰国正大国际商业广场、泰邦生物科技园、104国道泰安绕城西线等一批重大项目前期工作，使之尽快具备开工条件。三是注重优化投资结构。今天的投资结构就是明天的产业结构。坚持有保有压、区别对待，综合运用核准、环评、要素供应等手段，加大对关键领域和薄弱环节的投资力度，严格控制“两高”和产能过剩行业投资扩张。全年规模以上投资预期1740亿元左右，其中社会民生、服务业、高新技术和战略性新兴产业、技术改造等领域1500亿元。四是强力招商引项目。进一步创新体制机制，创新招商方式，突出产业招商，在招大引强上下功夫。实行产业招商责任制，建立产业、部门、园区、企业相结合的招商体系，强化对重点项目的调度、检查、考核，努力推进产业招商实现大的突破。特别是对重点招商项目，实行一个项目、一名包保领导、一个责任单位、一套工作班子，确保一抓到底，尽快投产。充分发挥企业招商的主体作用，积极推进委托招商、以商招商、园区招商、会展招商、境外招商。认真研究后金融危机时代产业转移和资本流动的规律，面向世界500强和国内100强特别是“央”字号大企业、大集团，实行定向招商、优中选资，力争引进一批大项目、好项目。加大对上争取力度，争取更多的项目和资金落户泰安。认真研究山东半岛蓝色经济区、黄河三角洲高效生态区以及省会城市群经济圈等省重点发展战略的有关政策，做好对接争取工作，突出抓好泰安高新区海洋产业配套服务、肥城现代盐化工等联动示范基地建设。认真落实促进民间投资的政策措施，激发民间投资热情。

　　(二)做强做大工业，夯实富民强市发展基础。工业是经济发展的台柱子，坚持以“双轮驱动”为抓手，推进工业做强做大。一是大力培育优势产业和骨干企业。以先进制造业为主攻方向，加快实施工业调整振兴意见和各专项规划，着力培育一批规模大、效益好、市场竞争力强的骨干企业和优势产业。突出抓好43个汽车产业项目、28个输变电产业项目和46个新材料项目，进一步凸显产业优势；以现有骨干优势企业培植壮大和引进大项目为途径，促进企业扩大规模，努力打造一批销售收入过百亿、过五百亿乃至上千亿的企业和企业集团，进一步增强综合竞争能力。二是改造提升传统产业。加快推进利用信息技术改造提升煤炭、化工、食品、纺织等传统产业，努力提高内在素质和竞争力。年内计划实施过千万元的技术改造项目422个，力争技改投资增长14%。重点抓好泰丰矿业20万吨碳酸二甲酯、岱银集团牛仔束染和特殊纱、瑞星集团煤制烯烃和尿素深加工等项目，构筑转型发展新优势。拓展技改的深度和广度，推动技改由设备更新为主向对生产全过程和产品结构调整的总体改造转变，由单个企业改造向整个产业链的配套改造发展，由单纯的生产制造环节改造向研发、设计、营销、服务等领域延伸。三是积极发展战略性新兴产业。千方百计加快新材料、新能源与节能环保、电子信息、生物医药与生物育种四大战略性新兴产业发展，突出技术和产品开发、产业化、市场培育等关键环节，扶持一批重点产业和骨干企业。全力推进泰山玻纤高强高模高性能玻璃纤维、宝来利来倍利素、华兴纺织纯壳聚糖纤维等列入省战略性新兴产业项目建设，争取早日竣工投产发挥效益。四是大力支持小微企业加快发展。全面落实支持小微企业发展的各项金融财税政策，实施结构性减税让利，规范税费征管，完善金融服务，帮助小微企业减负增效、稳健经营、健康发展。充分发挥新型工业化产业示范基地引领作用，鼓励中小企业主动承接大企业零部件配套业务，完善、拉长产业链，促进特色产业聚集壮大。五是深入开展“品牌建设年”活动。大力实施质量兴市战略，培植一批名优品牌，促进工业经济发展从“资源依赖”向“科技依托”、“泰安制造”向“泰安创造”转变。

　　(三)大力发展旅游文化产业，推动服务业提速增效。围绕把旅游业培育成经济发展的战略性支柱产业，把泰安建设成为山东旅游文化产业高地，深入推进旅游、文化、服务业间的融合发展，促进整体上档次、上规模、上水平。一是推动旅游业快速高效发展。积极推进旅游管理体制改革创新，强化政府主导，实施部门联动，着力整合资源，尽快实现由依赖泰山向依靠泰山多点联动转变、由观光旅游向观光休闲度假旅游转变、由单一旅游发展向旅游文化融合发展转变，构建大泰山大旅游大产业格局。积极推进特色美食街、高铁游客集散中心等服务设施建设，完善城市旅游服务功能。加大旅游促销力度，推进服务标准化建设，打造“好客泰安”旅游服务品牌。二是大力推进旅游与文化融合发展。充分发挥文化旅游的叠加效应，以文化项目建设和文化企业发展为切入点，把文化资源变为旅游景观，以旅游景观彰显独特厚重的泰山文化，努力提升泰安、泰山旅游的品位和档次。年内，重点抓好泰山文化产业园、泰山千古情城、太阳部落、泰山温泉国际会议中心、瑞奥不夜城、泰安文化艺术中心、泰山玉石市场等项目建设，着力培育一批有核心竞争力的文化龙头企业，不断提高旅游文化产业专业化、规模化和集约化水平。进一步放宽民营企业文化市场准入，完善金融支持民营文化企业发展的办法，加快培育文化要素市场，构建统一开放、竞争有序的文化市场体系。三是进一步加快服务业发展。坚持服务业协调推进机制，促进服务业拓宽领域、优化结构、提升层次。重点抓好现代物流、科技、信息、金融、职业教育与培训等生产性服务业，改造提升商贸、餐饮、房地产、家庭及社区服务等生活性服务业，着力培育节能环保、工业设计、健康服务、服务外包、会展服务、汽车服务等新兴服务业，积极发展农村服务业，促进区域城乡服务业协调发展。加大服务业载体建设扶持力度，对10个服务业重点园区、30个重点企业和30个重点项目加强调度管理，培植壮大一批拥有自主知识产权和知名品牌、具有较强市场竞争力的服务业龙头企业。

　　(四)加大三农工作力度，加快现代农业建设进程。坚持把解决“三农”问题作为各项工作的重中之重，进一步落实强农惠农富农政策，促进农业持续增效、农民持续增收、农村持续稳定。一是确保粮食生产安全。实施好国家新增千亿斤粮食生产能力基地县建设工程，深入开展优质粮食高产创建活动。认真落实农业补贴政策，确保新增农业补贴向种养大户、农民专业合作社倾斜，加快推进农业机械化、规模化、产业化。二是大力发展特色优势农业。全面落实粮食、蔬菜、奶牛、花卉苗木、泰山茶等14个农业产业振兴方案和年度计划，努力培育一批有市场竞争力和影响力的农产品品牌，力争高效特色农业产值占农业总产值的比重提高1个百分点以上。三是以工业化理念推进农业现代化。按照产业化、规模化、标准化、现代化的要求，紧紧围绕主导产业和优势农产品精深加工，延伸产业链条，加大扶持力度，支持骨干龙头企业做强做大。大力推进农产品质量安全检验检测体系工程建设，建立健全农产品标准体系、安全检测监管体系和标准认证体系，推进农业标准化生产。大力发展各类农民专业合作组织，推动农业家庭经营向农户联合与合作多元化、多层次、多形式经营体系转变。四是加大农业科技创新力度。有效整合科技资源，推动产学研、农科教紧密结合，健全完善现代农业产业技术体系。加强农业科技的推广应用和创新服务，启动公益性农业科技专项，搞好现代农业示范区建设，打造农业技术示范和推广平台。加强对农民的技术培训，不断提高农民的科技素质和科技应用创新能力。五是拓宽农民持续增收途径。大力发展农村二、三产业，鼓励农民自主创业，扩大农村劳动力转移就业，努力增加农民非农收入和工资性收入。抓好农民负担监督管理，防止农民负担反弹。六是加强农村综合生产能力建设。加强农田水利基础设施建设，配套完善灌溉体系，推进节水灌溉，努力实现“耕地灌区化、灌区节水化、节水长效化”。抓好天泽湖、汶口二号坝工程建设。组织实施水系生态建设规划，抓好小流域综合治理。深入实施农村饮水安全工程，搞好水土流失治理和造林绿化，严格保护耕地，抓好农村土地综合整治，加强农业综合开发，努力提高农业的综合效益和持续发展能力。

　　(五)完善城镇综合服务功能，不断提高城镇化建设水平。按照《泰安市城市总体规划(2011-2020年)》要求，编制完善道路交通、绿地水系、公共服务设施、城市色彩及风貌特色等专项规划，强化规划的龙头和调控作用。搞好各类基础设施配套建设，加快“两轴”、“两带”、高铁新区、长城路南段、旅游经济开发区、东部文化旅游区、南部城市新区等重点片区开发建设，年内重点抓好龙潭路景观提升、温泉路北段建设、南关路南段大修、建筑垃圾综合处置场建设、虎山公园扩建、城中村改造、长城路南段综合开发建设、七里河综合整治、中央公园建设、岱庙周边改造等工程，整体提升配套水平和城市功能。落实“公交优先”政策，优化站点布局，实行延时服务，畅通泰城交通，保证居民和游客进出方便。以“争创中国人居环境奖”为目标，深入开展城乡环境综合整治，推进城中村集中连片改造，探索建立城市管理的长效机制，优化改善城市环境，提升市民居住生活质量。充分发挥县城、中心镇、农村社区带动区域经济发展的作用，实施城乡规划、建设、产业一体化，逐步推进城乡共建、城乡联网、城乡共享。搞好国家重点镇和省级中心镇扩权强镇试点工作，扎实推进以城边村、园区周边村、经济强村、矿区压煤村为重点的新型农村社区建设。认真实施《泰安生态市建设总体规划》，建立一批自然保护区、重要生态功能保护区；围绕预防、预警和应急三大环节，构建突发环境事件和气象、地质、生物等灾害安全防控体系；加大创建国家环保模范城市力度，争取今年通过国家验收；抓好绿色低碳重点小城镇建设，努力改善城乡人居环境，促进人与自然和谐发展。

　　(六)深化改革开放，增强经济与社会发展的活力与动力。改革开放是发展的必由之路、强市之路，必须坚持不懈地深入推进。一是深入推进文化体制改革，加快国有经营性文化单位转企改制步伐，推动文艺院团走向市场。积极推进非时政类报刊改革，加大资源整合力度，办好《泰山晚报》，做大做强泰山报业集团，推进广播电视产业化。二是加快财政金融体制机制改革，创新完善政府投融资、财政管理、公共服务供给等机制，提升财政保障水平。规范政府融资平台，加强政企银合作。鼓励企业采用股票上市、再融资、发行债券等方式，拓宽直接融资渠道，重点做好众诚公司、力博科技、科诺型钢等15家企业上市培植工作，力争尽快上市。抓好6家小额贷款公司和11家融资性担保公司监管与协调服务，争取再设立1-2家小额贷款公司。继续推进农村信用社改革，支持符合条件的市县联社组建农村合作银行。抓好3家已签署协议的村镇银行的设立，争取实现村镇银行县域全覆盖。促进民间信贷规范化发展，依法打击非法集资等违法犯罪行为，防范金融风险。三是加快资源性产品价格的市场化改革，健全污染者付费、生态补偿制度，建立健全资源环境产权交易机制。四是深化国资国企改革，进一步健全组织管理体系，扩大监管范围，落实监管职责，加强资本运营，增强国有企业的活力和效率，促进国有资产保值增值。五是深化农村改革，在稳定土地承包关系的基础上，加快建立健全土地流转市场，引导土地承包经营权规范有序流转，发展多种形式的适度规模经营，提高农业的规模效益。加快供销社改革发展，扎实推进国有林场国家级改革试点。六是深化综合配套改革，支持试点单位先行先试、创新发展模式。

　　进一步提高对外开放水平。坚持高层推进对外开放，以项目建设为中心，以招商引资为手段，以“两市(京沪)、两地(港台)、两国(日韩)、两洲(欧美)”为主攻方向，努力提高利用外资水平。立足现有企业，加大对外贸易培训力度，不断扩大外贸企业队伍，深度开拓多元国际市场，努力扩大出口规模；继续加强出口名牌产品培育和出口示范基地建设，加快外贸转型升级步伐。积极开展对外工程承包，规范外派劳务。拓展外向型经济的广度和深度，构筑全方位对外开放格局。扎实推进对口支援新疆和扶贫协作重庆等项工作。

　　(七)着力提升自主创新能力，大力推进节能减排。坚持把自主创新作为转方式、调结构的中心环节，大力发挥科技、人才、管理等生产要素作用，努力促进经济增长由主要依靠增加物质消耗向主要依靠科技进步、劳动者素质提高、管理创新转变。一是强化自主创新。加大财政科技投入，建立以政府投入为引导、企业投入为主体、社会资金广泛参与的科技投入体系。加快推进重点高新技术产业壮大、战略性新兴产业培育、农业科技带动、社会发展领域科技支撑、科技创新体系建设推进、知识产权战略提升等六大工程建设。优化配置各类科技资源，加强重点领域和关键技术的研究，开展产学研联合，加强创新平台建设；培育一批拥有自主知识产权的创新型企业和知名品牌。支持企业承担国家、省级重大科技项目，以科研基地和创新项目为依托，提升企业自主研发能力，增强核心竞争力。大力实施人才强市战略，着力引进和培养高层次、实用型、创新型人才。二是强力推进节能减排。突出抓好重点行业和企业的节能改造，扶持一批节能技术项目，继续淘汰一批落后产能，坚决限制高能耗产业发展。坚持实施水污染、大气污染、固体废物污染综合治理，把主要污染物排放总量削减指标纳入各级政府目标管理，实行严格的污染物达标排放控制标准。加强污染专项整治，搞好污水管网铺设和节点连通。强力推进节约集约用地，提高土地利用效率。大力发展绿色经济、循环经济和新型低碳产业，积极推进大唐东平、国电新泰、华电肥城等新能源项目建设。全面实施《泰安市用水总量控制管理实施办法》，实行“三条红线”管理，落实最严格的水资源管理制度，加快推进节水型社会建设。

　　(八)繁荣发展社会各项事业，加强社会管理创新。以社会管理创新试点工作为抓手，协调发展各项社会事业，努力构建既充满活力又和谐稳定的新泰安。一是坚持教育优先发展战略。认真组织实施中长期教育改革和发展行动计划，加大对教育事业的投入，提高财政教育支出占财政支出的比例，确保达到省核定标准。实施学前教育三年行动计划，大力推进公办幼儿园建设。实施县域义务教育均衡发展规划，加快推进普通中小学办学条件标准化建设、中小学校舍安全、农村初中校舍改造等工程。搞好农村义务教育学生营养改善。高度重视并加强校车安全和校园治安工作。大力发展职业教育，培养更多更好的技能型人才。支持驻泰高校改革与发展，充分发挥高校支持地方经济社会发展的作用。二是大力发展卫生事业。深化医药卫生体制改革，推进公立医院综合改革试点工作，加快基层医疗卫生机构综合配套改革，把村卫生室和非政府办基层医疗卫生机构逐步纳入基本药物制度实施范围，全面落实基本药物配备使用、医保报销和对乡村医生的补偿政策。加强医疗卫生机构建设，优化完善城乡基层疾病防控、医疗保健、卫生监督和应急体系。扶持发展中医药事业，提升中医药服务能力。三是繁荣发展文化等社会事业。完善文化政策保障机制，大力发展公益性文化事业，抓好重点公共文化设施建设，深入实施文化惠民工程，加大广播电视村村通、乡镇(街道)综合文化站、农村文化大院、公共电子阅览室、农家书屋、农村公益电影放映等建设工作力度，切实改善基层文化条件，保障人民基本文化权益。加强对文化遗产的保护和利用。强化人口和计划生育目标管理责任制，切实稳定低生育水平，积极推进统筹解决人口问题。加强体育基础设施建设，广泛开展全民健身活动，提升竞技体育水平。依法加强宗教事务管理，认真落实少数民族政策，扶持发展少数民族经济。坚持普惠制度和特惠制度相结合，加快构建与经济社会发展水平相适应的残疾人社会保障和服务体系。重视抓好国防动员、人民防空、民兵预备役、双拥共建工作，积极争创新一轮国家双拥模范城。关心支持老龄、妇女儿童事业发展，加强气象防灾和人工影响天气工作，做好外事侨务和史志、档案、防震减灾等项工作。

　　加强和创新社会管理。继续推进社会管理创新综合试点工作，巩固提升成果，努力提高社会管理整体水平。深化“平安泰安”建设，强化社会治安综合治理，高度重视人民来信来访，更加注重从源头上预防和减少社会矛盾，维护社会和谐安定。深入开展“六五”普法和“五五”依法治市，提高全社会自觉学法、守法、用法意识，夯实社会和谐的法制基础。加强食品药品基层监管站所和电子监管信息化建设，强化重大疫病防控，加大行政执法和责任追究力度。深入开展“企业主体责任落实年”活动，落实安全生产责任，提高安全监督水平，加强对矿山、化工、交通、森林、公共场所等重点行业、重点领域的安全生产专项整治，坚决遏制重特大安全事故。加快建设社会信用体系，建立健全覆盖全社会的征信系统，加大对守信行为鼓励保护和对失信行为的惩戒力度。

　　四、以民为本，建设人民群众满意的政府

　　面对新形势、新任务，政府及其工作人员必须进一步增强责任感和紧迫感，坚持以民为本、富民为责、勤廉为要的理念，切实加强自身建设。

　　高度重视改善民生。把保障和改善民生放在更加突出的位置，完善政策、增加投入，多办惠及民生、改善民生的实事。坚持就业优先战略，深入实施更加积极的就业政策，千方百计扩大就业容量，提高就业质量，确保就业局势稳定。抓好重点群体就业工作，继续将促进高校毕业生就业放在首位，统筹做好农村劳动力、城乡就业困难人员和部队退役人员就业。强化就业指导，加强职业培训，提高就业服务水平，着力破解“就业难”、“招工难”两难并存问题。进一步完善覆盖城乡的社会保障体系，努力扩大社会保障覆盖面，增强保障能力，提升保障水平。巩固城乡居民社会养老保险全覆盖成果，基本解决未参保集体企业退休人员纳入基本养老保险遗留问题，完善养老、医疗、失业、工伤、生育保险市级统筹。继续增加企业退休人员养老金。将新型农村合作医疗和城镇居民医疗保险政府补助标准提高到每人每年240元，逐步提高新农合和城镇基本医疗保险最高支付限额和住院费报销比例。提高城乡居民低保和优抚对象生活补助，重度残疾人生活补贴标准由每人每月50元提高到70元。抓好保障性住房建设，多渠道积极筹措建设资金，加大财政支持力度，全面完成省下达我市的保障房建设任务。完善保障住房分配管理办法和租赁补贴制度，确保分配公平、管理规范、群众满意。结合新型农村社区建设和生态文明乡村建设，推进农房建设工作常态化。

　　努力加强政府自身建设。一要努力学习。牢固树立终身学习的理念，全面系统地学习中国特色社会主义理论体系，尤其重视加强对科学发展观、转方式调结构等重大理论和决策的学习，加强对现代科技、金融知识、法律、市场经济和履行岗位职责所需专业知识的学习，优化知识结构，提升工作境界。要笃学深思，学以致用，学用结合，使学习成果转化为谋划发展的思路、促进工作的措施、解决问题的能力，不断增强领导科学发展的本领。二要加快政府职能转变。深化行政审批制度改革，进一步理顺政府与企业、市场、社会的关系，充分发挥市场配置资源的基础性作用，着力推进政企分开、政资分开、政事分开、政府与市场中介组织分开，为经济发展创造良好环境。加快推进政府管理创新，整合集约利用行政服务资源，实施政务服务信息化，简化办事程序，提高工作效能。加强政府门户网站、公共行政服务中心、便民电话受理中心等服务窗口建设，切实提升公共服务水平。三要大力加强作风建设。加强领导干部作风建设，引导和督促各级领导干部树立正确的政绩观，履职尽责，埋头苦干。强化服务意识，提高行政服务质量和效率，降低企业和基层获取政务服务的成本。坚持问政于民、问需于民、问计于民，真诚倾听群众呼声，真实反映群众愿望，真情关心群众疾苦，切实维护人民群众合法权益。深入基层、深入群众，加强对群众最盼、最急、最忧、最怨问题的调查研究，找准要害、完善措施、解决问题。弘扬求真务实作风，认真治理慵懒散问题，严肃处理不作为、乱作为等行为。四要坚持依法行政。坚持依法科学民主决策，重大事项集体决策，健全决策程序，规范行政行为，强化决策责任。健全完善执法责任、效能监察、政务公开、举报投诉制度，确保权力依法公正运行，始终接受监督。坚持向人大报告工作和向政协通报工作制度，认真执行人大及其常委会的决议、决定，自觉接受人大的法律监督和政协的民主监督，认真办理人大代表议案、建议和政协提案。加强同各民主党派、工商联、人民团体和社会各界人士的联系。高度重视网络舆情，主动了解包括“微博”在内的社情民意。五要清正廉洁。自觉遵守党的纪律和廉洁自律的各项规定，做到立身不忘做人之本，为政不移公仆之心，用权不谋一己之私，修身自省，为政清廉。坚持从严施政，加强制度建设和监督制约，严格按照法定权限和程序行使好权力、履行好职责。积极开展行风评议、行风热线和专项治理等活动，强化对权力的监督和领导干部经济责任审计，加大查办违纪违法案件力度。大力弘扬艰苦奋斗精神，厉行勤俭节约，把心思、精力和财力用在发展经济、改善民生上，提高人民群众的满意度。

　　各位代表，泰安的发展繁荣，为百姓谋福祉，是我们崇高的追求，责任比泰山还重。让我们高举邓小平理论和“三个代表”重要思想伟大旗帜，更加紧密团结在以胡锦涛同志为总书记的党中央周围，在中共泰安市委的坚强领导下，认真贯彻落实科学发展观，锐意进取，真抓实干，团结拼搏，努力保持经济平稳较快发展和社会和谐稳定，谱写推进富民强市、建设幸福泰安的新篇章，以优异成绩迎接党的十八大胜利召开！
